# Children at risk of domestic accidents when are locked up at home: the other side of COVID-19 outbreak lockdown

**DOI:** 10.1186/s13052-022-01318-2

**Published:** 2022-07-27

**Authors:** Valentina Ferro, Raffaella Nacca, Mara Pisani, Sebastian Cristaldi, Maria Francesca Faa, Maria Chiara Supino, Umberto Raucci, Antonino Reale, Marta Ciofi Degli Atti, Massimiliano Raponi, Alberto Villani, Anna Maria Musolino

**Affiliations:** 1 Department of Emergency and General Pediatrics, Bambino Gesù Children’s Hospital, IRCCS, Rome, Italy; 2 Clinical Pathways and Epidemiology Unit-Medical Direction, Bambino Gesù Children’s Hospital, IRCCS, Rome, Italy; 3 Medical Direction, Bambino Gesù Children’s Hospital, IRCCS, Rome, Italy

**Keywords:** Home accidents, COVID-19, Lockdown, Children, Emergency

## Abstract

**Background:**

We proposed to analyze thoroughly the impact of the COVID-19 lockdown (CL) in changes of profiles and in trend of the domestic accidents (DAs) in children.

**Methods:**

This was a single experience, cross-sectional study conducted at the emergency department (ED) of III trauma center. We enrolled children under 18 years admitted to ED with a diagnosis of DAs comparing the CL period from 10^th^ March 2020 to 4^th^ May 2020 with the same period of the previous year,10^th^ March 2019 to 4^th^ May 2019.

**Results:**

In CL period, the cumulative incidence of ED visits for DAs increased from 86.88 to 272.13 per 1,000 children and the cumulative incidence of hospitalizations for DAs increased from 409.72 to 534.48 per 1,000 children. We reported in CL a decrease in the severity of ED presentation assessed by proxy measures: the level of priority ED visits reduced by 67% in CL period (OR: 0.33; 95%CI 0.22–0.48; *p* < 0.001); the likelihood of delayed time of presentation to ED increased by 65% in case of domestic injuries occurring in CL period (OR: 1.65; 95% CI: 1.17–2.34; *p* = 0.004); the odds of transfer from other hospital decreased by 78% in CL (OR: 0.15–0.33; *p* < 0.001). Children were more at risk of poisoning (OR:3.35–106.11; *p* = 0.001), of body foreign ingestion (OR: 1.83–14.39; *p* = 0.002) and less at risk of animal bite trauma (OR:0.05–0.35; *p* < 0.001).

**Conclusion:**

Although the need to stay home has made a decisive breakthrough on the spread of COVID-19, the experience from this study underlines how this preventive measure has also had a downside in term of increased cumulative incidence of ED visits and of hospitalizations for DA.

## Introduction

The COVID-19 pandemic outbreak induced Italian Government, as well as many countries in the world, to adopt an emergency protocol for containing and mitigating spread of SARS-CoV-2. On10^th^ March 2020 the lockdown had beginning and for the first time after the World War II a curfew was established in addition to the closure of shops, except those selling crucial necessities, the cessation of sport, of cultural manifestations and of any kind of public or private event, the closure of schools and universities, the ban on leaving home or moving except for medical purposes and proved needs.

Despite children have been relatively spared during the first wave of the pandemic, with a low number of reported infections, of hospitalization rates and of mortality [1] with a reduction of presentation to emergency department (ED) and healthcare use [2, 3], the lockdown had harmful collateral effects on life and wellbeing of children and young people extending beyond those of direct viral infection. In particular, it is interesting that some studies have evaluated the incidence of childhood domestic accidents (DAs) during the COVID-19 lockdown (CL) period, but these presented a variability of results in term of increase or decrease as well as the injury characteristics and mechanism [4–8]. However, many of these studies comprised sporting injuries as well as motor vehicle occupant or pedestrian injuries.

There are poor study analyzing exclusively the trend of the child DAs in CL period in Italy that has been the first European country to experience the spread of the Sars-Cov2 virus. Previously, only *Bressan et al*selected only accidents sustained in the household environment, but in this case, they only described the comparison of pediatric ED presentations and hospitalizations for DAs overall and by four injury categories - trauma, poisoning, burn, and foreign bodies (FBs) - during the COVID-19 outbreak lockdown and the corresponding period of the previous year [7].

Based on this, we proposed to analyze thoroughly the impact of lockdown in changes of profiles and in trend of the DAs in children in comparison with the corresponding period of the previous year.

## Materials and methods

This was a retrospective, cross-sectional study conducted at the ED of III trauma center, in Rome, Italy. The study was approved by the Ethics Committee of our institution according to the Declaration of Helsinki (as revised in Seoul, Korea, October 2008). The number protocol was 2210_OPBG_2020.

We enrolled children aged to < 18 years admitted to the ED with a diagnosis of DAs that we defined as “any event occurring inside the house or in immediate surroundings of house that resulted in injury” [9] which was not done deliberately but happened by injury.

We compared the CL period from 10^th^ March 2020 to 4^th^ May 2020 with the same period of the previous year, 10^th^ March 2019 to 4^th^ May 2019.We excluded the intentional or abusive trauma and self-inflicted injuries and patients with missing data.

We collected demographic data, injury characteristics (mechanism, pattern, injured body region) and clinical management (medical disposition for hospitalization, need for diagnostic investigation and/or specialistic consultation at ED, need for treatment in hospital and follow up) for all patients with DAs from electronic chart at ED.

We researched both the label “domestic accident” in the panel of the patient records on informatic regional system for the management of EDs in Lazio region and the description of the event in the clinical history of each patient reported by pediatricians.

At the admission of each child in our ED, the priority of visits was assigned in accordance with clinical condition severity and in line with the regional guidelines: the need of immediate-intermediate medical care versus low-non urgent medical care. We defined the delayed time of ED presentation if the injury was occurred ≥ 24 hours, while the admission to ED was classified as transferred and direct. These three variables were considered as proxy indicators of the severity of ED presentations.

The circumstances and mechanism of injury were coded according to The International Classification of External Causes of Injury (ICECI) [10].

The primary outcome was the epidemiological trend of DAs during the study period. The secondary outcomes were the demographics of the people injured, the injury context and characteristic (i.e., the type of injury, anatomical location, and the rates of hospitalization, treatment and follow up).

Due to retrospective design, we enrolled all patients with a diagnosis of DAs, and therefore, we estimated the power of the study. Considering the prevalence of the DAs in our ED in 2019 equal to 8.7% and in 2020 equal to 27% and the overall population of patients admitted to ED in 2019 (10,117) and in 2020 (3,649) and considering the Confidence Interval (two-sided) of 95, the power of the study was 100%.

### Data analysis

A Statistical analysis was performed using the software STATA/IC 14.2 version 2017. We tested the normality by Skewness/Kurtosis test. Data were reported as median values with an interquartile range (IQR) and direct comparisons were made with Mann-Whitney rank-sum tests. Percentages were used to describe categorical outcomes and distributions of categorical data were compared with either a Pearson’s χ^2^ test or a Fisher’s exact test, as appropriate.

A multivariable logistic regression was performed to identify independent risk factors for DAs associated with CL period. Variables were chosen based on the univariate significance.

We reported their point estimates (adjusted odds ratio [OR] for sex and age) together with their 95% confidence intervals (CIs). Statistical significance was taken at *P* less than 0.05.

**Fig. 1:**
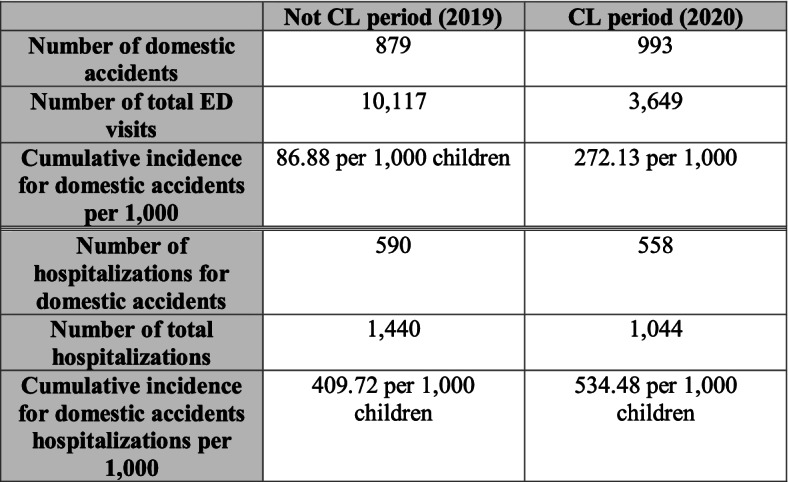
Comparison of ED visits and hospitalizations for domestic accidents, during the COVID-19 outbreak lockdown and the same period of the previous year

In addition, we calculated the cumulative incidence of visits for DAs in children during both from 10^th^ March 2020 to 4^th^ April 2020 and the same period in the previous year 10^th^ March 2019 to 4^th^ April 2019 by dividing the number of injured participants by the number of children admitted to ED over the specified period (Figure 1). We also calculated the cumulative incidence of hospitalizations for DAs in the specified period (Figure 1).

## Results

A total of 1,872 ED visits for DAs were identified across the two periods: 879 in the year 2019 cohort and 993 in the 2020 lockdown one. During not-CL period, we registered 10,117 total ED visits and 1,440 children were hospitalized. In this period, the cumulative incidence of visits for DAs in children at our ED during the specified period was 86.88 per 1,000 children, and the cumulative incidence of hospitalizations for DAs was 409.72 per 1,000 children (Figure 1). During CL period, we reported 3,649 total ED visits and 1,044 children were hospitalized. The cumulative incidence of ED visits for DAs in children at our ED during the specified period was 272.13 per 1,000 children while the cumulative incidence of hospitalizations for DAs was 534.48 per 1,000 children (Figure 1). However, the number of hospitalizations was 61.36% with a significant decrease in lockdown period (from 67.20% to 56.19%; *p*<0.001) (Table 1).

**Table 1:** Comparison of characteristics of domestic accidents during the COVID-19 outbreak lockdown and the same period of the previous year

Characteristics	Total = 1,872	2019 *n* = 879	2020 *n* = 993	*P value*
Sex, n(%)				
• Female	821(43.86)	371(42.21)	450 (45.32)	0.176
• Male	1,051 (56.14)	508 (57.79)	543 (54.68)	
Age, (months), median (IQR)	54 (25–102)	64 (23–120)	50(26–86)	< 0.001
Age groups, n(%)				
• Infant and toddler (0 to < 3 years)	675 (36.06)	305 (34.70)	370 (37.26)	< 0.001
• Preschooler (3 to < 6 years)	469 (25.05)	174 (19.80)	295 (29.71)	
• Child school (6 to 9 years)	313(16.72)	139 (15.81)	174 (17.52)	
• Adolescent (> 9 years)	415 (22.17)	261 (29.69)	154 (15.51)	
Ethnic group, n(%)				
• Italians	1,759 (94.01)	814 (92.71)	945 (95.17)	0.025
• Others	112 (5.99)	64 (7.29)	48 (4.83)	
Priority ED visits, n(%)				
• Emergency-Urgent	428 (22.86)	313 (35.61)	115 (11.58)	< 0.001
• Not urgent-Delayed	1,444 (77.14)	566 (64.39)	878 (88.42)	
Admission to ED, n(%)				
• Transferred from other hospital	433 (23.14)	350 (39.82)	83 (8.37)	< 0.001
• Direct	1,438 (76.86)	529 (60.18)	909 (91.63)	
Time of injury, n(%)				
• 8:00 AM-5:00 PM	643 (41.56)	371 (55.54)	454 (54.63)	0.151
• 5:00 PM-12 PM	825 (55.04)	268 (40.12)	355 (42.72)	
• 12PM-8AM	51 (3.40)	29 (4.34)	22 (2.65)	
Delayed time of presentation to ED, n(%)	364 (19.45)	134 (15.26)	230 (23.16)	< 0.001
Injury mechanism, n(%)				
Fall,	1,286(68.70)	591 (67.24)	695 (69.99)	0.2
Collision	163 (8.71)	76 (8.65)	87 (8.76)	0.930
Stretching/traction	129(6.89)	74 (8.42)	55 (5.54)	0.014
Crushing	53(2.83)	23 (2.62)	30 (3.02)	0.60
Burn	10 (0.53)	2 (0.23)	8 (0.81)	0.09
Poisoning	40 (2.14)	2 (0.23)	38 (3.83)	< 0.001
Foreign body ingestion	40 (2.14)	6 (0.68)	34 (3.43)	< 0.001
Foreign body inhalation	1 (0.06)	1 (0.11)	0	0.470
Foreign in nose, ear, genital tract	53 (2.83)	15 (1.71)	38 (3.83)	0.006
Electrocution	0	0	0	0
Drowning	0	0	0	0
Bite injuries caused by domestic animals	97 (5.18)	86 (9.78)	11 (1.11)	< 0.001
Injury pattern, n (%)				
Soft tissues (muscles, tendons, and ligaments) contusion	212 (11.32)	78 (8.87)	134 (13.49)	0.002
Trauma of parenchymal organs	19 (1.01)	13 (1.48)	6 (0.60)	0.06
Fracture	769 (41.10)	411 (47.9)	348 (35.05)	< 0.001
Sprain/dislocation/strain	219 (11.70)	142 (16.15)	77 (7.75)	< 0.001
Wounds: cut/scrape/ abrasion/ scratch/laceration/erosion/ulceration/bruise	452 (24.14)	219 (24.9)	233 (23.49)	0.452
Head trauma	384 (20.51)	131 (14.90)	253(25.48)	< 0.001
Other injuries	15 (0.80)	13 (1.48)	2 (0.20)	0.002
Body region, n(%)				
Upper extremities	709 (37.87)	353 (40.16)	356 (35.85)	0.06
Lower extremities	267 (14.26)	144 (16.38)	123 (12.39)	0.014
Maxillofacial area	219 (11.70)	60 (6.83)	159 (16.01)	< 0.001
Trunk	82 (4.38)	39 (4.44)	43 (4.33)	0.906
Neck	201 (10.74)	193 (21.96)	8 (80.81)	< 0.001
Head	394 (21.05)	149 (16.95)	245 (24.67)	< 0.001
Clinical management				
Need for hospitalization, n(%)	1,148 (61.36)	590 (67.20)	558 (56.19)	< 0.001
Need for diagnostic investigation/specialistic consultation at ED, n(%)	1,420 (75.85)	627 (71.33)	793 (79.86)	< 0.001
Need for treatment, n(%)	1,215 (64.94)	539 (61.39)	676 (68.08)	0.002
Need for follow up, n(%)	939 (50.16)	499 (56.77)	440 (44.31)	< 0.001

The demographic, epidemiological and clinical characteristic of the total population and the populations of the two groups are presented in Table 1. The median age at admission was significantly higher in the 2019 [64 months (23-120) vs 50 months (26-86); *p*<0.001]. The adolescents were more prone to DAs in 2019 than in 2020 (29.69% versus 15.51%), while the preschoolers presented more frequently DAS in 2020 than 2019 (29.71% versus 19.80%).

The number of patients who applied with a high priority indicator for ED visits was significantly reduced, going from 35.61% in 2019 to 11.58% in the CL period, as well as the number of patients transferred from other hospital was significantly reduced in 2020 period compared with the previous one (39.82% versus 8.37%; *p*<0.001). The frequency of delayed ED presentation increased significantly from 15.26% to 23.16% in CL period (*p*<0.001).

When evaluating the mechanism of injury, we noted the frequency of poisoning, FB ingestion, and introduction of FB in nose, ear, and genital tract increased significantly in the CL period (respectively from 0.23% to 3.83%; 0.68% to3.43%; 1.71% to 3.83%); instead, animal bite injuries decreased significantly from 9.78% to 1.11% in CL period.

The fracture was the most common pattern overall in both periods accounting for 41.1% but we also reported a significant reduction of cases in CL period (from 47.9% to 35.05%; *p*<0.001).

The CL cohort presented a higher number of diagnostic investigation and/or specialist consultation. The number of DAs needing treatment in hospital increased significantly in CL cohort, but the need for follow up decreased in the CL cohort as well (see Table 1).

### Multivariable logistic analysis

The multivariable model (Table 2) shows that the odds of DAs in CL period decreased by 1% with each one month increase in age in CL period compared with previous period (OR: 0.99; 95%CI 0.990-0.997; *p*<0.001).

**Table 2:** Logistic multivariable regression exploring the relationship between the characteristics of domestic accidents the COVID-19 outbreak lockdown

2020 vs 2019	OR	Std. Er	z	P > z	95% CI	
					**Min**	**Max**
**Sex**						
Female vs Male	1.0002	0.13	0.02	0.986	0.77	1.30
**Age** (months)	0.99	0.0016	-4.12	< 0.001	0.990	0.997
**Ethic groups**						
Italians vs others	1.24	0.34	0.8	0.426	0.73	2.11
**Priority ED visits**						
Emergency-Urgent vs Not urgent-delayed	0.33	0.06	-5.73	< 0.001	0.22	0.48
**Admission to ED**						
Transferred vs direct	0.22	0.04	-7.64	< 0.001	0.15	0.33
**Delayed time of presentation to ED**						
(≥ 24 h)	1.65	0.29	2.85	0.004	1.17	2.34
**Injury mechanism**						
** Stretching/traction**	0.38	0.13	-2.86	0.004	0.19	0.74
** Poisoning**	18.84	16.62	3.33	0.001	3.35	106.11
** Foreign body ingestion**	5.13	2.70	3.11	0.002	1.83	14.39
** Foreign in noise, ear, genital tract**	2.11	0.83	1.9	0.06	0.98	4.55
** Bite injuries caused by domestic animals**	0.13	0.07	-4.06	< 0.001	0.05	0.35
**Injury type**						
** Fracture**	0.39	0.11	-3.4	0.001	0.22	0.67
** Sprain/dislocation/strain**	0.66	0.20	-1.35	0.176	0.36	1.20
** Soft tissues (muscles, tendons, and ligaments) contusion**	1.69	0.39	2.23	0.026	1.07	2.67
** Head trauma**	1.76	0.60	1.66	0.096	0.90	3.44
**Body region**						
** Other injuries**	0.06	0.05	-3.32	0.001	0.01	0.32
** Lower extremities**	0.49	0.10	-3.69	< 0.001	0.33	0.71
** Maxillofacial area**	1.99	0.49	2.82	0.005	1.23	3.22
** Neck**	0.003	0.0015	-11.82	< 0.001	0.0012	0.008
** Head**	1.09	0.37	0.25	0.802	0.56	2.13
**Clinical management**						
** Need for hospitalization**	0.09	0.02	-12.09	< 0.001	0.06	0.13
** Need for specialistic consultation or/and diagnostic investigation at ED**	2.98	0.61	5.31	< 0.001	1.99	4.45
** Need for treatment**	3.89	0.86	6.16	< 0.001	2.53	6.00
** Need for follow up**	0.22	0.06	-5.81	< 0.001	0.14	0.37
** Constant**	14.18	6.06	6.21	< 0.001	6.14	32.76

*OR* Odds Ratio

*Std. Er* Standard errors

*95% CI* 95% confidence interval for the OR

The level of priority ED visits reduced by 67% in CL period (OR: 0.33; 95%CI 0.22-0.48; *p*<0.001), while the likelihood of delayed time of presentation to ED increased by 65% in case of DAs occurring in CL period (OR: 1.65; 95% CI: 1.17-2.34; *p*=0.004). The odds of transfer from other hospital decreased by 78% in CL (OR: 0.15-0.33; *p*<0.001)

While the odds of the animal bite injuries decreased (OR: 0.13; 95% CI 0.05-0.35; *p*<0.001), the odds of poisoning and FB ingestion were over 18 and over 5 times, respectively, as higher in CL period (OR: 18.84; 95% CI3.35-106.11; *p*=0.001 and OR: 5.13: 95% CI 1.83-14.39; *p*=0.002).

We found the likelihood of fracture in CL period decreased (OR: 39;95%CI 0.22-0.67; *p*=0.001) but the likelihood of soft tissues contusion increased (OR: 1.69; 95% CI 1.07-2.67; *p*=0.026).

The odds of hospitalization decreased by 91% in lockdown (OR: 0.09; 95%CI: 0.06-0.13; p<0.001) as well as the need for follow up decreased by 78% (OR: 0.22; 95%CI: 0.14-0.37; *p*<0.001). However, children reporting a DA in CL were almost three times likely to have a specialistic consultation and/or diagnostic investigation at ED than 2019 period (OR: 2.98; 95%CG: 1.99-4.45; *p*< 0.001). Likewise, DAs in CL period were almost three times more likely to need a treatment than 2019 period.

## Discussion

Our study describes the changes in the epidemiologic profile of DAs in pediatric population at ED during COVID-19 pandemic lockdown. Overall, the most interesting results were the increase of the cumulative incidence for ED visits for DAs in children, and despite the decrease of the number of hospitalizations, we also registered an increase of the cumulative incidence of hospitalizations in CL period. However, the severity of injury presentations at ED decreased as it was evidenced by the decrease in the number of patients who applied for a higher indicator for priority ED visits, by the reducing frequency of patients transferred to our trauma center from other hospitals, and by the increase in the frequency of delayed ED presentation in CL.

The increasing trend of the cumulative incidence for ED visits for DAs in children was aligned to other studies [7, 8, 11, 12] but in contrast with others [5, 6, 13, 14]. These discrepancies might be attributed to different research methodologies (i.e., the different period compared with the lockdown period, the inclusion of all injury types comprising recreational or sport activities related, the geographic areas, the degree of adherence to restrictions in different countries). This increase might be explained as a psychological consequences of COVID-19 home confinement, in fact children were forced to spend more time at home and, in this circumstance, they were physically less active, depressed, and bored, unable to have in-person contact with their classmates, friends and teachers, and suffered for the lack of personal space [15, 16]. Dealing with the home confinement is a particularly stressful experience for parents who must balance personal life, work, and raising children being left alone without other resources with a potentially cascading effect on children’s wellbeing [17]. Therefore, the detrimental health effects of the home confinement combined with the addition of home environment stressors may have contributed to a fractured family support structure, decrease child supervision, and increase trauma [11] and all these aspects might explain the younger age of children having DAs. In fact, during lockdown, older children, now staying at home, spent more time playing videogames, watching TV, or following lessons online, because of the ban on team sports and the limiting of outdoor activity, and we know that the sporting injuries are usually sustained by older children [18].

As it has just been asserted, in our study the severity of injury presentations at ED decreased as evidenced by the decrease in the rate of patients who applied with a higher indicator for priority ED visits. This finding was going to add up the decrease of patients transferred to our trauma center from other hospitals, and the increasing frequency of the delayed ED presentation in CL. The delayed presentation or the avoidance of the ED during the COVID-19 pandemic might be explained by the parental concerns about their children to contract the infection while attending medical setting or the parental hesitancy to attend ED only in case of severe health conditions. We used these three variables - the priority of the ED visit, the transfer from other hospitals and the delayed ED presentation - as proxy indicators of the severity presentation at ED although, being not a class of direct indicators, their use can constitute a methodological limitation. However, no injury severity indicator is perfect and *Kim et al*highlighted that hospitalization and length of stay are often used as proxy measures of injury severity because they are routinely available in administrative datasets. Anyway, they are measures influenced by non-clinical factors such as gender and socioeconomic status, at difference of standardized triage score assigned to all patients attending ED [19].

The impact of home confinement has also influenced the injury pattern mechanism. In fact, although the fall represents the most typical mechanism in children under 5 years old [20], in CL period we noted that children were more likely to report poisoning or FB ingestion. This was expected, as above mentioned, considering the younger age of children in CL as previously reported [21, 22].

Instead, the risk of animal bite injury significantly was lower in CL than not-CL period. *Dixon et al*, for first, described an increase of dog bites from March to May 2020 during the confinement in the USA [23], while *Parente et al*reported a decrease in the number of children bitten in lockdown. However, authors specified that the lower frequency was due to the number of stranger dog bites that was drastically reduced, and stranger dog bites typically occur on the street or at the parks and playgrounds, places children have been less exposed to due to confinement [24].

Analyzing the injury types, the risk of fracture and other orthopedical lesions were significantly lower in CL. This data finding was expected considering that the most common mechanism of DAs occurring in CL were poisoning and FB ingestion, whereas fractures in pediatric population are generally due principally to fall caused by recreational and sport activities [25]. There are other studies showing a reduction in fractures [6, 13, 14].

Children reporting DAs during CL period were less likely to be hospitalized but the cumulative incidence of hospitalizations for domestic injuries*,*resulting more higher than not-CL period. This is due to the fact than the number of hospitalizations in relation to the total number of ED visits resulted increased in CL. This difference with the not-CL was probably attributed to the change of our internal hospital protocol for injury treatment at ED. In fact, before COVD-19 outbreak the injury needing an operative procedure could be performed directly at ED with the procedural sedation and analgesia (PSA) savings from avoiding unnecessary hospitalizations [26]. Many patients (based on clinical condition) could be directly discharged form ED after therapeutic procedure discharge criteria were satisfied. Throughout the world, this pandemic has brought about major changes in protocols and management strategies, therefore this protocol has been put on standby, and patients, needing operative procedure, were treated on an inpatient basis. Another alternative or concomitant explanation can be given by different types of DA arrived at the ED during the CL period or by the selection, made by the parents, of DA to be brought to the attention of the ED. This explanation might also affect the number of children needing treatment in hospital and specialistic consultation or/and diagnostic investigation at ED that was significantly higher in LC period than not-CL period. 

The limitations of the study include different aspects. The results reflect the experience of a single tertiary pediatric referral trauma center, and it may not be possible to extrapolate this to the actual incidence of DAs the population during the pandemic period.

We did calculate a severity score considering the variety of injuries, therefore we adopted the as a proxy indicator the priority for ED visits, the transfer from other hospitals and the delayed presentation to ED. We did consider preexisting conditions of fragilities or underlying diseases that could affect the injury risk as confounding factors. Finally, a limitation was on the collection of retrospective data of the database, therefore some adjustment factors were missing. For example, we did not gather data on the amount of time spent at home by children in relation to the incidence of DA and the incidence of DA in educational agency during the period 2019, which in an adjusted analysis would have given more meaningful data.

## Conclusions

This study shows that lockdown has influenced the epidemiological and clinical profiles of DAs at ED. In fact, although the executive prevention campaign called “Stay at Home, Stay Safe “was a successful and life-saving strategy that was inevitable during the first wave of pandemic, our study underlines how this preventive measure has also had a downside in term of increased cumulative incidence of ED visits and of hospitalizations for DA.

## Data Availability

The datasets analyzed during the current study are available from the corresponding author on reasonable request.

## Abbreviations

CL: COVID-19 lockdown
DAs: Domestic accidents
ED: Emergency department
FB: Foreign body
OR: Odds ratio
CI: Confidence interval
